# Robotic rectal cancer surgery in obese patients may lead to better short-term outcomes when compared to laparoscopy: a comparative propensity scored match study

**DOI:** 10.1007/s00384-018-3030-x

**Published:** 2018-03-25

**Authors:** Sofoklis Panteleimonitis, Oliver Pickering, Hassan Abbas, Mick Harper, Ngianga Kandala, Nuno Figueiredo, Tahseen Qureshi, Amjad Parvaiz

**Affiliations:** 1 Poole Hospital NHS Trust, Longfleet road, Poole, BH15 2JB UK; 2 School of Health Sciences and Social Work, University of Portsmouth, James Watson West, 2 King Richard 1st road, Portsmouth, PO1 2FR UK; 3 Champalimaud Foundation, Av. Brasilia, 1400-038 Lisbon, Portugal; 4 Bournemouth University School of Health and Social Care, Bournemouth, UK

**Keywords:** Robotic, Laparoscopic, Rectal cancer surgery, Obese

## Abstract

**Purpose:**

Laparoscopic rectal surgery in obese patients is technically challenging. The technological advantages of robotic instruments can help overcome some of those challenges, but whether this translates to superior short-term outcomes is largely unknown. The aim of this study is to compare the short-term surgical outcomes of obese (BMI ≥ 30) robotic and laparoscopic rectal cancer surgery patients.

**Methods:**

All consecutive obese patients receiving laparoscopic and robotic rectal cancer resection surgery from three centres, two from the UK and one from Portugal, between 2006 and 2017 were identified from prospectively collated databases. Robotic surgery patients were propensity score matched with laparoscopic patients for ASA grade, neoadjuvant radiotherapy and pathological T stage. Their short-term outcomes were examined.

**Results:**

A total of 222 patients were identified (63 robotic, 159 laparoscopic). The 63 patients who received robotic surgery were matched with 61 laparoscopic patients. Cohort characteristics were similar between the two groups. In the robotic group, operative time was longer (260 vs 215 min; *p* = 0.000), but length of stay was shorter (6 vs 8 days; *p* = 0.014), and thirty-day readmission rate was lower (6.3% vs 19.7%; *p* = 0.033).

**Conclusions:**

In this study population, robotic rectal surgery in obese patients resulted in a shorter length of stay and lower 30-day readmission rate but longer operative time when compared to laparoscopic surgery. Robotic rectal surgery in the obese may be associated with a quicker post-operative recovery and reduced morbidity profile. Larger-scale multi-centre prospective observational studies are required to validate these results.

## Introduction

Laparoscopic colorectal surgery has become the new standard for colorectal diseases as it offers several advantages over open surgery such as shorter hospital stay, earlier return to normal function, less postoperative pain, early mobilisation and improved cosmesis [1–6]. There is consensus amongst surgeons that obesity increases the technical difficulty of colorectal surgery [7, 8]. Obese patients tend to have a thickened and excessive omentum and mesentery which restricts the space for instrumental manoeuvre, limits access and vision, distorts the surgical planes and can lead to problematic bleeding [9]. With obesity becoming increasingly a major hazard to public health worldwide, colorectal surgeons are likely to encounter and operate on this group of patients in increasing numbers [10, 11].

Laparoscopic rectal surgery is particularly demanding, with studies reporting high conversion rates for rectal surgery and two recent multi-centre randomised control trials (ALaCaRT and ACOSOG Z6051) failing to show that laparoscopic surgery is non-inferior to open surgery [4, 12–14]. This is because existing laparoscopic instruments have a restricted range of movement compared with that of the surgeons hand and are difficult to use in confined spaces such as the bony pelvis [15, 16]. These limitations are magnified in the obese due to the even greater restriction of available space for manoeuvre in this group of patients.

Robotic surgical systems were designed to overcome the limitations of laparoscopic surgery by providing stable three-dimensional views from a surgeon-controlled camera, angulated instruments with 7° of freedom, markedly improved ergonomics and tremor filtering [15, 17]. These advantages are particularly attractive in rectal surgery, overcoming the challenges of laparoscopic instrumentation and technique in obese patients, where space is limited and accurate dissection in narrow deep cavities is required. However, whether robotic rectal surgery in the obese translates to superior short-term surgical outcomes is hitherto poorly examined. At present, there are only two studies comparing the short-term surgical outcomes of obese patients having laparoscopic and robotic rectal surgery [18, 19], with both studies including smaller sample sizes.

The aim of this study is to build upon current evidence by analysing and comparing the short-term surgical outcomes of obese patients undergoing robotic and laparoscopic rectal resection surgery.

## Methods

A retrospective analysis of prospectively maintained databases was conducted for this study. Consecutive patients from three centres, two from the UK and one from Portugal, who received minimally invasive rectal cancer surgical resections between 2006 and 2017 were identified. The inclusion criteria included all obese patients (defined as patients with a BMI ≥ 30) receiving laparoscopic or robotic elective rectal resection surgery. The robotic cases were propensity score matched to laparoscopic cases to obtain comparable patients.

All cancer patients were discussed in a multidisciplinary team meeting (MDT) prior to initiating any type of treatment. Magnetic resonance imaging (MRI) scans were reported using the Mercury criteria, and preoperative chemoradiotherapy was given to patients with high risk for local recurrence (threatened circumferential resection margin ≤ 2 mm or T4 in staging MRI). Neo-adjuvant radiotherapy was not used where rectal cancers were considered resectable by total mesorectal excision (TME) with a likelihood of clear margins.

No specific selection criteria were used to allocate patients to laparoscopic or robotic surgery. Applied surgical modality was based on equipment and theatre availability. The robotic approach was the preferred approach following the adoption of robotic surgery in each unit. This was from May 2013 (Unit A), November 2015 (Unit B) and May 2016 (Unit C) for each unit, respectively. All recruited patients signed an informed consent form allowing their data to be used for analysis and its publishing. The requirements for anonymization of personal dataset by the Data Protection Act 1998 were satisfied. According to the Health Research Authority (HRA), this study did not require their approval due to its status as a clinical audit.

Patients included in the study had surgery performed by fully trained laparoscopic and robotic colorectal surgeons. The surgeon from one of the centres (surgeon AP) led a supervised training programme involving the remaining surgeons participating in this study, ensuring that the same standardised modular approach was used throughout. Data collection commenced when the participating surgeons began working in their respective units, between 2006 and 2012 for the UK centres and 2013 for the Portuguese centre. In addition, 15 robotic cases performed by surgeon AP in other centres as part of demonstrating/proctoring cases were included in the study.

### Surgical technique

A previously described, standardised modular approach was used for laparoscopic surgery [20, 21]. Medial to lateral colonic mobilisation with isolation of the main vessels using clips was applied followed by TME using monopolar diathermy [20, 21].

Robotic rectal resections were performed using a single docking fully robotic approach [22–24]. The da Vinci Si was used in one of the UK centres and the da Vinci Xi coupled with table motion in the remaining two centres. The principle of standardised technique developed for laparoscopic surgery was also used for robotic surgery. Procedures commenced with medial to lateral dissection followed by vascular control by ligating the main vessels. A three-step approach was used for splenic flexure mobilisation [25]. Patients with upper rectal tumours (10–15 cm from anal verge) received partial mesorectal excisions (PME), while all patients with tumours below 10 cm (i.e. for mid- and low-rectal tumours) received TMEs. TME was performed in the same stepwise manner as in the laparoscopic group, starting with posterior mobilisation followed by right lateral, anterior and left lateral mobilisation in a stepwise manner. All patients receiving complete TME surgery were given pre-operative bowel preparation the day before surgery and had loop ileostomies fashioned in cases where an anastomosis was formed. Postoperatively, all patients were managed using the enhanced recovery program described by Kehlet and Wilmore [26]. Patients were discharged home according to set criteria for discharge.

### Data collection and outcome assessment

The cohort characteristics and short-term surgical outcomes of obese patients receiving elective robotic and laparoscopic rectal surgery were analysed. Cohort characteristics analysed included age, BMI, gender, American Society of Anaesthesiologist (ASA) grade, neoadjuvant radiotherapy, operation performed and pathological T stage. Peri-operative data included operative time, estimated blood loss and conversion to open (defined as any incision needed to either mobilise the colon or rectum or ligate the vessels). Post-operative clinical data examined included length of stay, 30-day readmission, 30-day reoperation, 30-day mortality and anastomotic leak. Pathological data examined included lymph node yield and circumferential resection margin (CRM) clearance.

### Statistical analysis

Once collated, cleaned and checked data was analysed using IBM SPSS version 24 (SPSS Inc., Chicago, IL, USA). The robotic cases were propensity scored matched to laparoscopic cases. The variables used to calculate the propensity score matching were as follows: neoadjuvant radiotherapy (yes vs no), ASA grade (I, II vs III, IV) and p T stage (T0–2 vs T3–4). Propensity scores were calculated via logistic regression analysis by applying the Propensity Score Matching function on SPSS version 24 with the match tolerance set to 0.4.

Non-parametric data was expressed as median with interquartile range and parametric data as mean with standard deviation. Cohort demographic and clinical characteristics were compared using χ^2^ test or Fishers exact test for categorical variables, Mann-Whitney *U* test for non-parametric continuous variables and *t* test for parametric continuous variables. *P* values of < 0.05 were considered statistically significant.

Finally, univariate binary logistic regression analysis was performed on all obese patients receiving elective minimally invasive rectal surgery to assess whether surgical approach (robotic or laparoscopic) affected morbidity and mortality (defined as the presence of any of the following outcomes: 30-day reoperation, 30-day readmission, anastomotic leak and 30-day mortality). Following this, a multivariate model was applied where surgical approach was adjusted for all clinically relevant variables including age, gender, neoadjuvant radiotherapy, ASA grade (I-II vs III-IV) and p T stage (T0–2 vs T3–4). The constant was included in the analysis model, and data is presented as odds ratio, 95% confidence interval and *p* value.

## Results

A total of 222 obese patients received elective minimally invasive rectal cancer resection surgery (63 robotic, 159 laparoscopic). The 63 robotic cases were propensity score matched with 61 laparoscopic cases to reduce the effect of confounding factors in the analysis.

### Cohort characteristics

There were no differences in any of the cohort characteristic data retrieved for the two cohorts as Table 1 shows. Patients were matched for ASA grade, neoadjuvant radiotherapy and p T stage.

**Table 1:** Cohort characteristics

	Robotic (*n* = 63)	Laparoscopic (*n* = 61)	*p* value
Mean age ± SD	65.80	67.25	0.469^t^
Median BMI (IQR)	32 (30–35.7)	32 (30–34)	0.372^m^
Gender			
• male	40 (63.5%)	41 (67.2%)	0.663^c^
• female	23 (36.5%)	20 (32.8%)	
ASA grade			
• I	0	1 (1.6%)	0.523^c^
• II	47 (75.8%)	43 (70.5%)	
• III	15 (24.2%)	17 (27.9%)	
Procedure			
• High anterior resection	12 (19%)	13 (21.3%)	0.842^c^
• Low anterior resection	42 (66.7%)	37 (60.7%)	
• APER	8 (12.7%)	10 (16.4%)	
• Hartman’s	1 (1.6%)	1 (1.6%)	
Neoadjuvant radiotherapy	24 (38.1%)	14 (23%)	0.067^c^
p T stage			
• 0	5 (7.9%)	2 (3.3%)	0.403^c^
• 1	4 (6.3%)	9 (14.8%)	
• 2	19 (30.2%)	21 (34.4%)	
• 3	33 (52.4%)	28 (45.9%)	
• 4	2 (3.2%)	1 (1.6%)	

*t t* test, *m* Mann-Whitney *U*, *c* Chi square, *SD* standard deviation, *BMI* body mass index, *IQR* interquartile range, *ASA* American Society of Anaesthesiology, *APER* abdominoperineal resection, *p T stage* pathological tumour stage

### Peri-operative characteristics and outcomes

The peri-operative characteristics of the two groups are summarised in Table 2. Median operation time was greater in the robotic cohort (260 vs 215 min; *p* = 0.000). Blood loss and conversion rate were similar between the two cohorts. There were only two conversions to open, both in the laparoscopic group. The first patient had high ventilation pressures and was unable to tolerate the Trendelenburg position and pneumoperitoneum meaning an early conversion was undertaken. The second patient provided a challenge in access and exposure due to excessive visceral fat.

**Table 2:** Peri-operative characteristics and outcomes

	Robotic (*n* = 63)	Laparoscopic (*n* = 61)	*p* value
Median operative time in minutes (IQR)	260 (214–310)	215 (192.5–252.5)	*0.000* ^m^
Median estimated blood loss in ml (IQR)	17.5 (10–20)	10 (0–40)	0.152^m^
Conversion to open	0	2 (3.3%)	0.240^f^

Italics statistically significant

*m* Mann-Whitney *U*, *f* Fisher’s exact test, *IQR* interquartile range

### Post-operative clinical and pathological outcomes

Length of stay was shorter (6 vs 8 days; *p* = 0.014) and 30-day readmission rate lower (6.3% vs 19.7%; *p* = 0.033) in the robotic surgery arm of this study. There were no differences in any of the remaining post-operative clinical data (length of stay, 30-day readmission rate, 30-day reoperation rate, anastomotic leak rate, 30-day mortality rate) or pathological outcomes (lymph node yield and R0 clearance) between the two cohorts as summarised in Table 3.

**Table 3:** Post-operative clinical and pathological outcomes

	Robotic (*n* = 63)	Laparoscopic (*n* = 61)	*p* value
Median length of stay in days (IQR)	6 (5–8)	8 (6–14)	*0.014* ^m^
30-day readmission	4 (6.3%)	12 (19.7%)	*0.033* ^f^
30-day reoperation	0	2 (3.3%)	0.240^f^
30-day mortality	0	0	
Anastomotic leak	1 (1.9%)	0	1.000^f^
Mean lymph node yield ± SD	17 (13–23.25)	16 (12–23.5)	0.639^m^
R0 clearance	61 (96.8%)	60 (98.4%)	1.000^f^

Italics statistically significant

*m* Mann-Whitney *U*, *c* Chi square, *f* Fisher’s exact test, *IQR* interquartile range, *SD* standard deviation

There were four readmissions in the robotic cohort, these were due to the following: a patient with a wound dehiscence, a patient with a urinary tract infection and two patients with ileus. In the laparoscopic group, there were 12 readmissions due to a variety of reasons including the following: two patients admitted with non-specific abdominal pain, a patient with an infected haematoma, four patients with ileus, three patients with wound infections, one of which had a wound dehiscence and two patients with high stoma outputs admitted with dehydration. There were only two reoperations, both in the laparoscopic group. Indications included a patient with a malfunctioning stoma and a patient with a wound dehiscence.

### Logistic regression analysis

Univariate logistic regression analysis of all 222 cases showed that surgical approach did not affect morbidity and mortality for the participants in this study, even though this did approach statistical significance (*p* = 0.051). This was still the case in multivariate analysis when other clinically relevant factors were adjusted for (age, gender, neoadjuvant radiotherapy, ASA grade, p T stage) as detailed in Table 4 (*p* = 0.072).

**Table 4:** Univariate and multivariate logistic regression for morbidity and mortality

		Univariate				Multivariate		
	OR	95% CI lower	95% CI upper	*p* value	OR	95% CI lower	95% CI upper	*p* value
Approach (rob vs lap)	2.698	0.996	7.305	0.051	2.651	0.917	7.659	0.072
Age	0.978	0.945	1.011	0.188	0.972	0.937	1.008	0.120
Gender (male vs female)	1.283	0.602	2.736	0.518	1.338	0.614	2.914	0.464
Neoadjuvant RT	1.722	0.570	5.203	0.335	1.238	0.377	4.071	0.725
ASA grade (I-II vs III-IV)	0.753	0.343	1.653	0.480	0.635	0.277	1.457	0.284
p T stage (T0–2 vs T3–4)	1.158	0.562	2.386	0.691	1.172	0.559	2.455	0.675

*OR* odds ratio, *CI* confidence interval, *RT* radiotherapy, *ASA* American Society of Anaesthesiology, *p T stage* pathological tumour stage

## Discussion

The effect of robotic rectal cancer surgery in offering superior short-term outcomes in obese patients when compared to laparoscopy is largely hitherto unknown. Several studies indicate that obesity is a risk factor for worse short-term surgical outcomes in laparoscopic colorectal surgery [27–29]. However, this is still a subject of debate, with numerous studies demonstrating no difference in short-term outcomes between laparoscopic obese and non-obese colorectal surgery patients [30, 31]. Nevertheless, the role of robotic colorectal surgery in obese patients has only been investigated in a handful of studies [9, 18, 19, 32, 33], with only two comparing the outcomes of obese robotic vs laparoscopic rectal surgery patients [18, 19]. The remaining three studies compared the outcomes of obese versus non-obese patients receiving robotic rectal surgery, with all three studies demonstrating no difference in short-term outcomes between obese and non-obese patients [9, 32, 33].

In the study presented here, both the length of stay and 30-day readmission rate were lower in patients receiving robotic surgery while operation time was longer. However, there were no differences in any of the remaining short-term surgical outcomes between robotic and laparoscopic rectal surgery. Furthermore, surgical approach, whether robotic or laparoscopic, was not found to affect morbidity and mortality. This study demonstrates that robotic rectal surgery in the obese is both safe and feasible with results suggesting a quicker recovery and better short-term readmission profile when compared to laparoscopic surgery in obese patients.

Robotic systems offer superior stable 3D views and ergonomic wristed instruments which are particularly useful when operating in confined spaces such as the pelvis, making the robotic platform especially attractive for rectal surgery. Obese patients tend to have increased intra-pelvic fat, further restricting access, room for manoeuvre and surgical field visibility. By further restricting the available space in the already narrow pelvis, robotic platforms seem ideally suited for this group of patients. This is supported by the initial results of the ROLARR trial (NCT01196000), a large randomised control trial comparing robotic to laparoscopic rectal surgery [34, 35]. Data from this trial was presented at the ASCRS and EAES conferences in June 2015, where conversion rates were lower in the robotic cohort in obese patients [36]. Considering this in conjunction with the high conversion and CRM positive rates in recent multi-centre randomised control trials investigating the role of laparoscopic rectal surgery [12, 13], the role of robotic rectal surgery in the obese patients clearly warrants further investigation.

In a similar study to ours, Gorgun et al. [18] compared 29 robotic with 27 laparoscopic obese rectal surgery patients. In this study, there was no statistically significant differences in operative time between the two groups, although operative times were longer in both cohorts compared to our results (rob vs lap: 329 vs 295 min; *p* = 0.13). Similarly, Shiomi et al. [19] compared 52 robotic with 30 laparoscopic obese rectal surgery cases and again found no difference in operative time (rob vs lap: 238 vs 252; *p* = 0.39). In our study, median operative time was 45 min longer in the robotic group. This may in part be explained by the inclusion of several cases where the surgeons and theatre teams were still in the earlier stages of their learning curve, with active training led by the senior surgeon (AP) being undertaken in a number of the included cases. While all consecutive cases are included in the robotic cohort (including all the initial cases at each unit), the laparoscopic cases are selected from a much larger pool spreading over a longer period.

Early studies involving robotic rectal surgery demonstrated that a significant contributor to the prolonged operation time in robotic surgery was due to the time it took to dock and undock the robot, which in part, may have been due to the relative inexperience of the surgeon and theatre staff who were still on the early stages of their learning curve [37–42]. More recent studies report equivalent operation times between robotic and laparoscopic rectal surgery [43–46] with some even demonstrating shorter operation times for robotic surgery [47–50]. Furthermore, the laser target system and improved design of the da Vinci Xi (the latest model by Intuitive Surgical) make docking easier and faster and increase the feasibility of the single docking approach. These features are likely to help further reduce robotic rectal surgery operative times [51].

Furthermore, our results show that the median length of stay was 2 days shorter, and 30-day readmission rate was lower in the robotic cohort. This could be secondary to lower post-operative morbidity. Morbidity was assessed in this study by analysing readmission, reoperation and anastomotic leak rates. However, minor post-operative complications were not included (Clavien-Dindo 1–2) as these were not reported in the datasets. It is postulated that minor complications (Clavien-Dindo 1–2) may be reflected in a prolonged hospital stay, which may explain why length of stay was higher in the laparoscopic group. In addition, readmission rates tend to be higher when length of stay is shorter, since patients that are discharged early are more likely to be readmitted with complications that were not picked up during their original hospital stay. Considering that both length of stay and readmission rate were lower in the robotic group, it is reasonable to suggest that this may be due to reduced surgical morbidity. This is supported by two recent studies published by the Cleveland clinic group who reported that length of stay, readmission rate and mortality effectively predict complications [52, 53]. Considering that mortality was equal between the two groups in our study, these studies strengthen our argument that the reduced readmission rate and length of stay in the robotic cohort are due to a lower surgical morbidity profile.

It is worth noting that both Gorgun et al*.* [18] and Shiomi et al. [19] also demonstrated a reduced length of stay in the robotic group when compared to the laparoscopic cohort (*Gordun* et al: 6 vs 8 days, *p* = 0.02; *Shiomi* et al: 7 vs 9 days, *p* < 0.001). Both studies found that length of stay was shorter by 2 days in the robotic group, with Shiomi et al. also demonstrating that blood loss and complication rates were lower in the robotic cohort (blood loss: 10.5 vs 34 ml, *p* = 0.002; complication rate: 9.6 vs 30%, *p* = 0.04). Considering the results of these studies in conjunction with our results, it is conceivable that robotic rectal surgery in the obese can lead to a quicker recovery and reduced morbidity when compared to laparoscopic surgery.

Notwithstanding operative time, length of stay and readmission rate, there were no other differences in the examined short-term outcomes. Furthermore, in logistic regression analysis, surgical approach (robotic or laparoscopic) was not found to affect morbidity and mortality. However, we should note that the *p* value neared statistical significance in both univariate and multivariate analysis (univariate *p* = 0.051, multivariate *p* = 0.072) when examining whether surgical approach affects morbidity and mortality (Table 4). In the absence of a power calculation being performed, we need to acknowledge the risk of a type 2 error, and therefore, the *p* value might have been significant if a higher number of patients were recruited.

Our results support the feasibility and safety of robotic rectal surgery in obese patients. In the robotic group, there was no conversion, 30-day mortality or reoperation, and the estimated blood loss was very low. Furthermore, regarding the short-term pathological results, the lymph node yield was acceptable and the CRM margin was negative (R0) in 96.8% of robotic cases, which is superior to that reported in the recent laparoscopic rectal surgery trials (ALaCaRT [12]: lap 93%, open 97%; ACOSOG Z6051 [13]: lap 87.9%, open 92.3%). Additionally, there is perception in the surgical community that prolonged operative times are associated with worse short-term outcomes [54], especially in obese patients due to the prolonged fixed Trendelenburg position. However, this was not the case in our study, and our results suggest that robotic rectal surgery in the obese can lead to similar short-term outcomes and a quicker post-operative recovery regardless of operative time.

The main strengths of our study are that data was collected from three centres from two different countries and is contemporary data, rather than data collected as part of a study that possibly includes an element of performance bias in surgical trials [55]. In addition, due to the propensity score matching, the two cohorts were evenly matched in terms of cohort characteristics, strengthening our results. Additionally, as far as we are aware, this study includes the largest sample size of its kind. Acknowledging its limitations, our study is retrospective in nature and does not report any functional or long-term data. We should also note that all the laparoscopic procedures pre-dated the robotic cases and this could introduce an element of bias in our results. However, we believe this is unlikely since the two surgeons with the longest laparoscopic colorectal practice had completed laparoscopic colorectal fellowships, were experienced laparoscopic surgeons from the outset of their practice and were both trainers for the National Training Programme for Laparoscopic Colorectal Surgery (LAPCO) in the UK [56]. Furthermore, the robotic cohort is more likely to be affected by the learning curve of the surgeons, since all initial cases are included. In addition, the laparoscopic cases underwent the same standardised enhanced recovery programme which was later applied to all the robotic cases. As a result, we believe that by standardising peri-operative care, both groups are comparable and peri-operative care is unlikely to act as a confounding factor when assessing hospital length of stay.

In summary, robotic rectal surgery in the obese could lead to a quicker recovery and improved morbidity profile when compared to laparoscopy, despite being associated with a longer operative time. Larger-scale multi-centre prospective observational studies are required to further investigate this topic. In addition, urogenital function and long-term oncological data need to be included in these studies to illuminate a more holistic comparison.
